# A universal severity classification for natural disasters

**DOI:** 10.1007/s11069-021-05106-9

**Published:** 2021-11-30

**Authors:** H. Jithamala Caldera, S. C. Wirasinghe

**Affiliations:** Department of Civil Engineering, University of Calgary, Calgary, Alberta Canada

**Keywords:** Universal disaster severity classification scheme, Global disaster severity scale, Universal standard severity index system, Extreme natural events, Disaster definitions, Impact assessment

## Abstract

The magnitude of a disaster’s severity cannot be easily assessed because there is no global method that provides real magnitudes of natural disaster severity levels. Therefore, a new universal severity classification scheme for natural disasters is developed and is supported by data. This universal system looks at the severity of disasters based on the most influential impact factor and gives a rating from zero to ten: Zero indicates no impact and ten is a worldwide devastation. This universal system is for all types of natural disasters, from lightning strikes to super-volcanic eruptions and everything in between, that occur anywhere in the world at any time. This novel universal severity classification system measures, describes, compares, rates, ranks, and categorizes impacts of disasters quantitatively and qualitatively. The severity index is useful to diverse stakeholder groups, including policy makers, governments, responders, and civilians, by providing clear definitions that help convey the severity levels or severity potential of a disaster. Therefore, this universal system is expected to avoid inconsistencies and to connect severity metrics to generate a clear perception of the degree of an emergency; the system is also expected to improve mutual communication among stakeholder groups. Consequently, the proposed universal system will generate a common communication platform and improve understanding of disaster risk, which aligns with the priority of the Sendai Framework for Disaster Risk Reduction 2015–2030. This research was completed prior to COVID-19, but the pandemic is briefly addressed in the discussion section.

## Introduction

One or more natural disasters occur on most days somewhere in the world causing immense hardship to living beings and major damage and losses. Natural disasters can be land based (e.g., earthquakes), water based (e.g., river floods), atmospheric (e.g., tornadoes), biological (e.g., pandemics), extraterrestrial based (e.g., comet strikes), or any combination of these (e.g., undersea earthquake and tsunami). Although these disasters are different, their impacts on humans and habitats are similar. All natural disasters can cause loss of life and damage to humans and their possessions, and they disturb people’s daily lives. However, it is difficult to express the level of severity caused by different types of natural disasters, in different countries, and in different time periods because there is no agreed upon terminology, no global standard communication platform, and no single common measurement for all types of natural disasters for all stakeholders that can estimate the total impact of an event and understand the full scope of severity. Further, there is no system that can be used for communication purposes without confusion and for educating the public regarding the disaster continuum.

Disasters do not respect national boundaries. Therefore, an international standard communication platform for severity is vital to have agreement among countries. The impact of a disaster in a region, if not managed properly, can produce political and social instability and affect international security and relations (Olsen et al. 2003). Agreed upon terminology in terms of quantifying “disaster” matters, inconsistencies in measuring disaster by stakeholders pose a challenge globally in terms of formulating legislation and policies responding to the disaster (Yew et al. 2019). There are no existing frameworks nor tools that holistically and objectively integrate all aspects of humanitarian need in terms of quantifying various natural disasters (Yew et al. 2019). Epidemiologic research of disasters is also hampered by a lack of uniformity and standardization in describing these extreme events (de Boer 1997). In addition, the foundation of any science is definition, classification, and measurement (de Boer 1990), and if disaster management is to grow and progress effectively, it also must have a consistent and recognized definition, classification, and measurement system.

Confusion occurs because the definitions of disaster terms in ordinary dictionaries are very wide, and different terms are used in different ways (Rutherford et al. 1983). For example, as stated in Definition and classification of disasters: introduction of a disaster severity scale (de Boer 1990), “it is difficult to evolve a meaningful definition of the word disaster. Most dictionaries identify this as a calamity or major accident and, while this is correct, such a definition fails to reveal why a calamity or major accident should be a disaster. From a medical point of view it is, therefore, of utmost importance to construct a simple definition for a disaster and, at the same time, to outline the criteria for its classification. Once such criteria have been determined, a scale can be evolved from which the gravity of the disaster can be assessed, which also allows the scientific comparison of various events.”

As a solution to the lack of uniformity and standardization in describing disaster events, numerous severity scales have been developed over the last three decades around the globe. These severity scales (quantitative, qualitative, or both quantitative and qualitative) are used for different purposes, by different stakeholder groups, and for all types of natural disasters (or particular types of disasters). Among the various scales with different measurement systems, a few common classifications for all types of natural disasters for all stakeholders exist, but they also have several deficiencies. For example, Disaster Scope, introduced by Gad-el-Hak (2008a), uses causalities and area affected to classify five severity levels (small, medium, large, enormous, and gargantuan disasters). Another by Eshghi and Larson (2008) uses fatalities and affected people to categorize six severity levels (emergency situation, crisis situation, minor disaster, moderate disasters, major disasters, and catastrophe). The ranges of Eshghi and Larson’s categorization supported by data, and the categories are determined using a statistical analysis of historical disasters, while the ranges of Disaster Scope are arbitrary. However, the proposed factors and their labeling appear to be arbitrary in both scales but are needed to conduct meaningful research. In addition, Disaster Scope and Eshghi and Larson’s classifications are highly related to vulnerability factors of a society and do not consider damage to humans possessions, such as cost of damage; therefore, the most expensive natural disasters that do not cause a severe loss of human life in a heavily populated area are not properly categorized compared to other disasters. Presently, no current scale identifies the relationship between severity and impact factors; therefore, there is no scientific instrument that supports the data and can clearly classify a disaster’s severity. Thus, attaining the real scope of a disaster's severity cannot be understood because no existing system consistently distinguishes the different severity levels.

Although there are many scales, clearly expressing the level of severity is difficult for two main reasons: first, there is no globally accepted standard to communicate the level of severity of a natural disaster (Caldera et al. 2016a), and second, there is no single measurement that can estimate the full scope of a disaster (Yew et al. 2019). Consequently, there is no common system to help emergency responders measure the impact of natural disasters, to determine the proper allocation of resources, and to expedite mitigation processes. Therefore, a nation’s ability to manage extreme events, including natural disasters and other perils, is difficult when there is no consistent method or mutual understanding among emergency management systems of different countries at all levels: international, continental, regional, national, provincial, and local. Therefore, a common severity classification system for all types of natural disasters for all stakeholder groups is required to understand, communicate, and educate the public on the nature of natural disasters.

This paper presents a universal severity classification scheme for all types of natural disasters that is applicable to all stakeholders, from civilians to responders to policymakers, to generate a common platform for expressing the impacts of disasters. This system will provide an overall picture of the severity of natural disasters, yield independent estimates of a disaster’s magnitude, help one to understand the disaster continuum, and assess a disaster for various purposes, such as helping governments and relief agencies respond when disaster strikes. In addition, the system is expected to gauge the need for regional, national, and international assistance and to help in communicating the severity of a disaster.

## Necessity of a universal severity classification

### Descriptive terms

Obtaining a sense of the real magnitude of a disaster’s severity is problematic and cannot be comprehended by merely using common descriptive terms because there are no consistent definitions, methods, nor a clear sense of scale to distinguish one term from another (Caldera et al. 2016a). To describe the severity level of a natural disaster, which can range from a small community fire to large-scale events such as a tsunami or earthquake, we often use words such as “emergency,” “disaster,” and “catastrophe.” The majority opinion is that a “disaster” refers to a large-scale emergency, and “catastrophe” refers to a large-scale disaster (Penuel et al. 2013). Though these words imply increasing levels of severity, one observer’s “disaster” might be another’s “catastrophe” depending on the experience, knowledge, and personal feelings toward the event. In the literature, there is controversy about whether the term “catastrophe” can be differentiated from “disaster” or whether they are synonyms (Penuel et al. 2013). Therefore, clear definitions and order of seriousness for the descriptive terms are important to categorize the severity of disasters.

### Levels of severity

It is common for events that have very different levels of severity to be put into the same category. For example, both the 1998 Hurricane Mitch (Schenk 1999) and the 2004 Indian Ocean Tsunami (WiscNews 2018) are categorized as a catastrophe. However, compared to the tsunami, Hurricane Mitch’s impact was much smaller: It struck 8 Caribbean and Central American countries and killed 11,000 people, while the Indian Ocean tsunami affected 12 countries of Asia and Africa and killed about 230,000 people. The root of this problem is that there is an insufficient number of categories representing the seriousness of a natural disaster; hence, using terms such as emergency, disaster, and catastrophe does not provide a sufficient level of detail to provide a clear understanding of the impact of an event.

Therefore, more levels are required to accurately categorize the impact of natural disasters. Determining the number of levels for all disasters and for all fields (e.g., medical field, rescue field, etc.) is not feasible. However, the confusion can be minimized when there is an adequate number of levels to distinguish between different categories of seriousness and when a consistent/standard number of levels exists.

### Objective measures

When describing a disaster, we not only use words, but also accompanying numbers. Natural events are often described using many objective factors of severity, e.g., deaths, injuries, and property damage. By comparing damage and fatalities, some disasters are labeled as the most expensive (e.g., Great East Japan Earthquake and Tsunami in 2011 and Hurricane Katrina in 2005) (Brink 2019) and some are labeled as the deadliest disasters (e.g., Indian Ocean Earthquake and Tsunami in 2004 and Haiti earthquake in 2010) (Pappas 2018; Ritchie and Roser 2014).

However, a statistical comparison of disaster impacts is a complex task because various factors present different insights into the level of severity of an event. For example, comparing the number of fatalities and the total cost of damage gives contrasting ideas as to the level of severity of the 2004 tsunami that struck Sri Lanka versus the 2013 flood that struck Southern Alberta, Canada. The 2013 Southern Alberta flood resulted in $5.7 billion in damage, 4 fatalities, and affected 100,000 people (but no injuries nor did it leave anyone homeless) (Centre 2013), while the 2004 tsunami caused $1.32 billion in damage, but it caused more than 35,000 fatalities and affected more than 1 million people (with 23,000 injuries and 48,000 left homeless) (Centre 2013). If one considers only fatalities, then the Sri Lankan tsunami appears more severe, and if one considers cost of damages, the Alberta flood appears more severe. There are many factors that can be considered when addressing the severity of an event. No current scale identifies the relationships between impact factors nor uses these relationships to estimate the overall severity of a disaster (Caldera 2017).

Therefore, comparing levels of impact for different types of disasters is challenging. Nevertheless, comparing key impact factors (such as fatalities, injuries, homeless, affected population, and cost of damage), as shown in the comparison of the Sri Lanka tsunami and Southern Alberta flood, provides a more complete comparative picture between two disasters and a more comprehensive idea of the extent of damage, rather than merely comparing one or two factors, such as fatalities and/or damage costs. This more complete picture helps disaster and insurance managers to estimate the true magnitude of a disaster's severity (Caldera and Wirasinghe 2014), which cannot be comprehended using current approaches. Current inconsistent identification of disaster impacts results in overcompensation or undercompensation in assigning resources for mitigation. Overcompensation may result in wasting resources, while undercompensation could increase the impact severity. Thus, a proper technique is required to compare statistics and rate natural disasters based on severity.

### Severity of different disaster types

Generally, natural disasters are described according to their intensity or magnitude. However, earthquakes that are measured on the Richter scale cannot be compared to hurricanes that are measured on the Saffir-Simpson scale because these scales use different measures that cannot be compared easily. Clearly, these individual scales are useful. For example, knowing the range of wind speeds in a hurricane, as provided by the Saffir-Simpson scale, allows people to estimate potential damage to people and property (Gad-el-Hak 2008a). Although some disasters, such as earthquakes and hurricanes, have rating scales to measure strength, some other disasters do not have systemized metrics. The disasters that do not have rating scales are assessed by geographical measures. Nevertheless, when an area is prone to two or more disasters (e.g., earthquakes, floods, cyclones, etc.), disaster management centers (DMCs) must assess the appropriate combinations of disasters (e.g., earthquakes and tsunamis, or cyclones, floods, and landslides, or thunderstorms and tornadoes) and decide which combinations are specific to the area being assessed. They must then rank the most likely individual disaster or combination that could occur in that area (Wickramaratne et al. 2012). For instance, the Calgary Emergency Management Agency releases a list of top 10 hazards and risks in Calgary (Wood 2016). After ranking the hazards, DMCs must assess the potential impacts of each likely individual/combination event and take actions (or make decisions) based on the potential combined impacts. These impact assessments, with their criticality over other combinations in the list, allow DMCs to appropriately allocate the required resources with some justifiable basis. However, impact assessments are complicated by different type of unrelated scales.

### Disaster warnings

Warning indications during an event should be given in plain language so that everyone can understand the seriousness of a coming disaster and the urgency of evacuation when required. In warning communications, the intensity of a disaster is commonly used as the measure of the destructive power because the intensity/magnitude is assumed to be the most meaningful to the general public. However, intensity/magnitude levels are not the best way to describe the severity level of a disaster because they are an indication only of the strength (i.e., hazard potential) but not the impact (i.e., vulnerability of a region). As shown in Table 1, the impacts of a disaster are not highly correlated to existing scales for volcanic eruptions, earthquakes, tsunamis, and tornadoes because the Pearson correlation coefficients for impact factors and intensity/magnitude scales are less than 0.5 (Colton 1974). The impact depends on where a disaster occurs: It can be quite different in a populated city compared to a rural area. For example, a small hailstorm can significantly impact a city if it affects humans and their vehicles and dwellings compared to a strong tornado that occurs in a forested area with a very small population. Thus, the highest intense/magnitude event may not necessarily be the most disastrous. Specifically, a considerable body of research presents data that indicate people often underestimate or ignore warnings for natural disasters and other low probability events (Camerer and Kunreuther 1989; Meyer 2006). Severe natural disasters are low probability, high consequence events. Therefore, a new approach is required to communicate the warnings issued by emergency management systems to the general public so that there is a mutual understanding between both parties.

**Table 1:** Correlation between intensity scales and impact factors

Disaster	Existing Scale	Fatalities	Injuries	Damage	House Destroyed	House Damaged	Missing
Volcano	VEI scale	0.33	0.39	0.09	0.33	–	0.45
Earthquake	Richter Scale	0.13	0.285	0.488	0.23	0.237	–
Tsunami	Intensity Scale	0.248	0.134	0.168	0.043	–	–
Tornado	EF Scale	0.339	0.366	0.32	–	–	–

### Unified scale

Currently, different stakeholder groups have their own scales with different measurement systems to assess a disaster according to their requirements. For example, disaster managers and emergency responders use incident management teams (IMTs)—typing (United 2020; Alberta 2020), medical personal use disaster severity scale (DSS) (de Boer 1997), database managers use Munich RE global loss database categorization (Löw and Wirtz 2010), insurance managers use catastrophe models (Grossi et al. 2005) and logit and hazard models (Lee and Urrutia 1996). There are several disadvantages with these existing systems. These scales have various levels (between 3 and 13) to distinguish the destructive capacity of an event using various factors. Thus, some scales have a limited number of categories. Also, some classification systems (e.g., catastrophe models) are even confidential to the respective organizations. Additionally, some classification systems are not scientific and have arbitrary grading systems (e.g., DSS). However, most scales use fatality a factor to differentiate severity levels, except for IMTs for emergency responders and disaster managers (because IMT uses both impact and management challenges associated with response and recovery to categorize disasters). These individual scales are useful for specific groups; however, different scales, which are not integrated, cannot be used to convey the level of severity of an event for all stakeholders.

When a disaster strikes, these disconnected systems make it even more difficult for stakeholders to communicate about the severity of the disaster. Therefore, confusion and misunderstanding can occur. For example, most of the North American emergency management agencies use IMTs’ as a way to classify all hazards and to assign a type number to the incident, in order to address response and recovery activities and the command and control infrastructure that is required to manage the logistical, fiscal, planning, operational, safety, and community issues related to local/regional/national emergencies, natural disasters, and public events (Alberta 2015). IMTs are "typed" according to the complexity of incidents they are capable of managing and are part of an incident command system as shown in Table 2. In particular, a Type 5 IMT can manage a small community fire; however, to manage a major flood may require a Type 4 or lower IMT. Confusion can arise whether Type 1 or Type 5 is the most critical. Hence, a universal system that integrates the existing systems is essential.

**Table 2:** Incident Management Teams—Typing (Government 2021)

Type	Level	Staffing	Deployment	Incident
Type 1	National/Provincial level	35–50 trained personnel	Deployed as a complete team with all ICS positions staffed	Large number of resources (500–1000), multiple operational periods
Type 2	National/Provincial level	25–35 trained personnel	Deployed as a complete team with Planning, Logistics and Fin/Admin staffed	Large number of resources (200–500) and multiple operational periods
Type 3	Provincial/Regional	10–30 trained personnel	Deployed as teams of 10–15 (depending on need)	Major and/or complex Incidents/Events
Type 4	Regional/Local	10–15 personnel	Deployed as a Team to Community or County EOC	Expanded Incidents/Events
Type 5	Local	10–15 personnel	Deployed as a Team to Community or County EOC	Incidents/Events contained in one Operational Period

ICS = Incident command systems; EOC = emergency operations center

In addition, when a disaster is first identified, emergency responders often do not know the full scope of the disaster. An event can quickly escalate from a routine emergency to a disaster, and then to a bigger disaster. The management challenges associated with response and recovery also increase as impacts escalate. During events when emergency responders communicate with other stakeholders, such as national/regional/local governments, relief agencies, non-governmental organizations (NGOs), and the media, they have no common classification system that provides a unified understanding of the level of severity of the event. Consequently, officials who are trying to understand the full impact of a disaster do not have a consistent scale that can provide a clear understanding of the potential hazard, and so they cannot alert other stakeholders, such as the general public, about the degree of severity.

Moreover, the type is assigned by internal personnel and can be subjective due to the level of experience and internal processes used. Decisions made can delay the adoption of appropriate actions needed to mitigate a disaster; in other words, assistance from international governments, NGO’s, relief agencies, and volunteer communities can be delayed. The consequences of failing to identify a potential hazard and failing to manage a disaster adequately can be significant. For example, regarding Hurricane Katrina, Tierney (2008) explained that “… devastating impacts were worsened by a sluggish and ineffective response by all levels of government and by a lack of leadership on the part of high-ranking federal government officials and others who were incapable of recognizing Katrina’s catastrophic potential, even after the storm made landfall.” Inconsistent and disconnected severity measures mean that either members of the general public may not clearly understand the degree of the emergency or that members of emergency management systems may not clearly understand the potential hazard. Hence, a common platform that can integrate these disconnected metrics for all stakeholders is necessary for clear communication and understanding without confusion.

Furthermore, nation’s ability to manage disasters is more effective when there is mutual understanding between countries and different emergency management systems at all levels: international, continental, regional, national, provincial, and local. The ability of countries to manage extreme events can be dependent on the system that they use. However, since countries use different systems to manage extreme events, either a universal understanding of the systems used by other countries or a global standard is required to better prepare and manage global disasters that affect more than one country. For instance, if there had been a universal system in 2004 when the Indian Ocean tsunami struck 12 countries in Asia and Africa, it may have saved thousands of lives. Thus, a new approach is needed to mobilize resources properly, make adjustments as necessary, and more correctly gauge the need for regional, national, or international assistance. Therefore, there is a mandate for a new system that integrates both measurement systems: management and severity.

## Creating a universal severity classification

### Universal disaster severity classification

A consistent scale is needed to understand the disaster continuum and to develop a platform for a reliable and transparent data management process that facilitates comparisons between different disasters (Gad-el-Hak 2008a; Löw and Wirtz 2010).

### Objectives

Developing a Universal Disaster Severity Classification Scheme (UDSCS) is necessary to solve the previously mentioned problems. This new universal system is expected to integrate all current measurement systems: impacts, management, and size. The UDSCS will connect the current measurement systems and provide a common communication platform that can be used to compare, quantitatively and qualitatively, and measure, describe, and categorize the impact of disasters for the general public and emergency responders. Therefore, the UDSCS is expected to avoid inconsistencies and, most importantly, to connect the severity metrics to generate a clear understanding of the degree of an emergency and potential hazard.

### Development

The 5 key steps to develop a UDSCS are addressed by the following two questions: How many levels are required to clearly differentiate the impact of natural disasters? How are these levels used to clearly distinguish the various degrees of natural disasters, both quantitatively and qualitatively?

1. Identify the most influential factors related to disaster severity.
2. Develop the foundation of the UDSCS in terms of (i) the number of levels and (ii) associated color coding.
3. Develop qualitative measures by clearly defining words that describe disasters.
4. Develop quantitative measures that are based on data and statistically robust.
5. Develop the UDSCS.

## Foundation of the UDSCS and qualitative scale

### Step 1: the most influential factors

What makes a disaster “large scale” is the number of people affected by it and/or the extent of the damaged infrastructure and geographical area involved (Gad-el-Hak 2008b). However, there are many factors that need to be considered when addressing the severity of an event.

The severity of natural disasters increases as the impact to humans and their possessions increases and the power and intensity of an event increases. In contrast, severity decreases the more a region is prepared for a disaster. Therefore, severity relates to all factors that can be grouped as follows:

- Socioeconomic factors that reflect impact to humans and their possessions: number of fatalities, injuries, missing persons, homeless persons, evacuees, people affected by the disaster, the cost of damage (damage to property, crops, and economic damage), etc.
- Strength-measuring factors that reflect the power and intensity of an event: magnitude, duration, speed, location, distance from disaster site to affected populated area(s), etc.
- Preparedness factors that reflect a region’s preparedness: available technology, resources, whether the area(s) could be evacuated before being affected, mitigation methods, response rate, etc.

More details about the above groups can be found in Analysis and classification of natural disasters (Caldera 2017).

A scale representing all factors is complex. However, no matter how prepared people are, where the disaster occurred, or how intense/powerful the disaster is, if people lose their belongings or loved ones in a natural disaster, their disutility mainly depends on what they have lost and not how they prepared for the disaster, the intense/powerful the disaster, nor where the disaster occurred. Therefore, the severity of an event directly relates to socioeconomic factors and indirectly relates to strength-measuring and preparedness factors. Hence, the severity of an event can be evaluated by measuring the negative impact of a disaster on people and infrastructure (Wickramaratne et al. 2012).

### Step 2: the foundation of the UDSCS

#### Step 2, part (i): Proposed number of levels for the severity spectrum

According to the previous step, a multidimensional severity scale should include a cross section of socioeconomic factors. These factors can be further sub-grouped into human factors (e.g., fatalities, injuries, missing persons, homeless persons, evacuees, and affected population) and damage factors (e.g., cost of damage, damage to property, crops, and economic damage). A 0–10 level system is proposed because very large ranges of almost all socioeconomic factors can be expressed within 11 levels using the log scale. The 11 levels of human factors (H), which range from 0 to 7.674 billion people (the world’s population, World 2019a) are shown in Column 2 in Table 3. The 11 levels of damage factors (D), which can range from 0 to United State Dollar (USD) 87.698 trillion (the maximum gross domestic product in 2019, World 2019b) are shown in Column 3 in Table 3. The severity of a natural disaster is measured by the adverse effects of the event on a community or an environment and not the severity of the event on an individual person. Therefore, the three ranges of damage factors, which are 1 ≤ D < USD 10, USD 10 ≤ D < USD 100, and USD 1,000 ≤ D < USD 10,000, can be grouped into 1 ≤ D < USD 10,000. Therefore, 0–10 levels representing socioeconomic factors (impacts to human and material damage) are considered in designing the UDSCS.

**Table 3:** Ranges of human and damage factors in 0–10 levels

	Human factors (H)	Damage factors (D)
0	1 < H	1 < D
1	1 ≤ H < 10	1 ≤ D < 10
		10 ≤ D < 100
		100 ≤ D < 1,000
		1,000 ≤ D < 10,000
2	10 ≤ H < 100	10,000 ≤ D < 100,000
3	100 ≤ H < 1,000	100,000 ≤ D < 1 M
4	1,000 ≤ H < 10,000	1 M ≤ D < 10 M
5	10,000 ≤ H < 100,000	10 B ≤ D < 100 M
6	100,000 ≤ H < 1 M	100 B ≤ D < 1 B
7	1 M ≤ H < 10 M	1 B ≤ D < 10 B
8	10 M ≤ H < 100 M	10 B ≤ D < 100 B
9	100 M ≤ H < 1 B	100 B ≤ D < 1 T
10	1 B ≤ H	1 T ≤ D

M = Million; B = Billion; T = Trillion

The numbering systems most used, for example the metric system of measurement, are based on 10. Systems based on 10 are easy to use and easily administered and scored. The 0–10 level severity scale is easy to remember as the levels increase by a power of 10. Also, it is easy to integrate the foundation of the UDSCS with quantitative and qualitative measures using 0–10 levels, as explained in the next two steps. Thus, UDSCS considers the severity of disasters based on the most influential impact factors, including socioeconomic factors, and gives a rating from 0 to 10: 0 being no impact and 10 being worldwide devastation. Therefore, defining the foundation of the UDSCS with 0–10 severity levels is well suited, meaningful, and easy to remember for users.

#### Step 2, part (ii): Proposed color coding for severity levels

Currently, there is no consistent method of color coding. Different fields have different color coding, and there are even different colors used within the same field. For example, the NOAA National Weather Service, Weather Prediction Center (Weather 2019) uses white, green, yellow, red, and purple for rainfall warning signals, while the Philippine Atmospheric, Geophysical and Astronomical Services Administration (Philippine 2020) uses red, orange, and yellow.

The UDSCS is used by many stakeholders, including policy makers, governments, responders, and civilians. Because this system is widely used, and these stakeholders are familiar with the global color coding of traffic signals, the same color coding system was selected with some modification. Blue was added, and yellow was chosen instead of amber because it is one of the 3 primary colors and has a specific name in all languages. Blue, dark green, light green, yellow, and dark yellow represent lesser severity levels. Black and purple were added, and along with red, they (red, dark red, light purple, dark purple, and black) represent higher severity levels. White is also added to indicate nondestructive events. The color codes that correspond to the 11 severity levels are shown in Table 4. Introducing color coding that correspond to levels of severity is important because it eliminates language barriers and confusion that could arise. Further, everyone, including those who are illiterate, can quickly understand the UDSCS because colors easily explain the seriousness of a disaster. Although words in different languages can be found to represent each level of severity, there will be some people working or involved in disaster recovery who cannot understand the local language. Therefore, color coding is an effective means of communication, and the UDSCS can be adapted to any language, country, society, or culture.

**Table 4:**
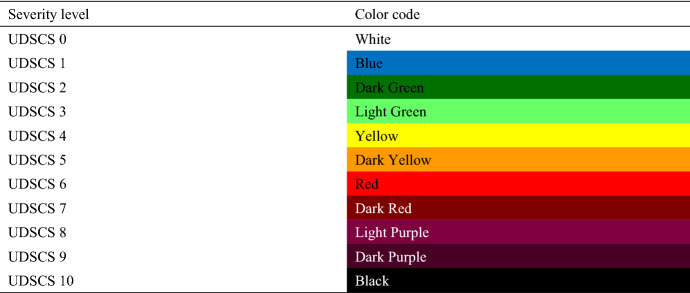
Levels and the corresponding color coding in the UDSCS

### Step 3: Developing qualitative measures

As a qualitative measure of the UDSCS, the linguistic method, which is the most commonly used and oldest method of describing natural disasters of various magnitudes, is used. For example, the words such as calamity, cataclysm, catastrophe, disaster, and emergency are used in this analysis to categorize the different levels of disaster impacts. Still, only words that describe the magnitude of the natural phenomena are considered. Therefore, words, such as “Armageddon,” which describe “a usually vast decisive conflict or confrontation” or “a terrible war that could destroy the world” (Oxford 2010) are not used because they do not refer to natural events but rather human-caused catastrophes.

However, the sense of the real magnitude of a disaster’s severity cannot be comprehended using the current linguistic method because it has several deficiencies described in the following subsection. Therefore, an order of seriousness and clear definitions for the considered terms are also proposed to describe the severity spectrum.

#### Deficiencies in the current qualitative measure

First, there are no consistent definitions, methods, or clear sense of scale to differentiate these terms (used to describe disasters) from each other. For example, the Oxford dictionary defines common terms used to describe disasters as follows (Oxford 2010):

- Apocalypse: an event involving destruction or damage on a catastrophic scale.
- Calamity: an event causing great and often sudden damage or distress; a disaster.
- Cataclysm: a large-scale and violent event in the natural world.
- Catastrophe: an event causing great and usually sudden damage or suffering; a disaster.
- Disaster: a sudden accident or a natural catastrophe that causes great damage or loss of life.
- Emergency: a serious, unexpected, and often dangerous situation requiring immediate action.

“Catastrophe” is used to define “disaster,” and “disaster” is used to describe both “catastrophe” and “calamity” in the Oxford dictionary (Oxford 2010), and therefore, definitions are circular, and words are used interchangeably to describe the seriousness and severity levels of natural events.

Second, vocabulary, context, and interpretation of each term is not fixed (Kelman 2008); therefore, the meanings of these words have changed over time. For instance, the first use of the word “disaster,” when it was first added to English vocabulary in the late sixteenth century, meant “ill-starred event” (Cresswell 2009), which implies an event affecting the planet to be in an ill state or destroyed. Currently, “disaster” is defined in the Oxford Dictionary as “a sudden accident or a natural catastrophe that causes great damage or loss of life” (Oxford 2010). Comparing the historical and current definitions shows how the meaning of these terms has changed over time. The etymological definitions of these terms are as follows (Oxford 2010):

- Apocalypse: uncover, disclose, reveal (late fourteenth century)
- Calamity: damage, loss, failure, misfortune, adversity (early fifteenth century)
- Cataclysm: to wash down (deluge, flood, inundation) (1630 s)
- Catastrophe: overturning, sudden turn (a sudden end) (1530 s)
- Disaster: ill-started event (the stars are against you) (1560 s)
- Emergency: to rise out or up (unforeseen occurrence requiring immediate attention) (1630 s)

Third, the order of seriousness implied by these terms has also changed over time because the meaning of these terms have changed. According to the word origins (Column 2, Table 5), and according to current English dictionary definitions (Column 3, Table 5), the order of seriousness of the terms from lowest to highest have changed, with the exception of “emergency,” which has remained at the same level over time (Caldera 2017). However, the term “emergency” currently describes different levels of severity, which is confusing. For example, government agencies use the term “emergency” to declare a state of emergency when there is a serious and uncontrollable situation. “Emergency” is also used to describe situations as small as a car accident. Therefore, “emergency” can be any level because it can describe situations as small as a car accident or as large as a major disaster. Therefore, the meaning of these words and the level of seriousness of each word should be fixed to clearly convey their implied severity level and to reduce confusion.

**Table 5:** Levels of seriousness of the terms according to historical and current dictionary definitions (Caldera 2017)

Seriousness	Historical	Current
Level 1	Emergency	Emergency
Level 2	Apocalypse	Disaster
Level 3	Calamity	Calamity
Level 4	Cataclysm	Cataclysm
Level 5	Catastrophe	Catastrophe
Level 6	Disaster	Apocalypse

Fourth, the meanings of the terms change depending on context, and according to the Oxford English Dictionary, there are many applications of the terms (Caldera 2017). For example, William Shakespeare used the term “catastrophe” to express an insult: “I’ll tickle your catastrophe” in Henry IV, Part 2 (Spevack 1973). However, “catastrophe” in geology is a sudden and violent change in the physical order of things, such as a sudden upheaval, depression, or convulsion affecting the Earth's surface and the living beings upon it. Some have supposed that a catastrophe occurs at the end of the successive geological periods (Oxford 2014). These terms are often used as metaphors and have different connotations. For instance, “disaster” can describe everything from an event like an earthquake to occasions when two ladies turn up for a party wearing the same dress (de Boer 1990).

Even within the same field, definitions of the descriptive terms vary. For instance, the EM-DAT database has defined “disaster” as a “situation or event, which overwhelms local capacity, necessitating a request to national or international level for external assistance; an unforeseen and often sudden event that causes great damage, destruction and human suffering. Though often caused by nature, disasters can have human origins” (EM-DAT 2021). Canada’s emergency management framework (3rd Edition) defines disasters as “Essentially a social phenomenon that results when a hazard intersects with a vulnerable community in a way that exceeds or overwhelms the community's ability to cope and may cause serious harm to the safety, health, welfare, property or environment of people; may be triggered by a naturally occurring phenomenon, which has its origins within the geophysical or biological environment or by human action or error, whether malicious or unintentional, including technological failures, accidents and terrorist acts” (Public 2017). The Encyclopedia of Crisis Management’s disaster classification, which is given in Table 6, has four levels (incidents, major incidents, disasters, and catastrophes) and outlines “disaster” according to impacts and management challenges of response and recovery (Penuel et al. 2013).

**Table 6:** Differentiation of the size of an event by process and impact (Penuel et al. 2013)

	Incidents	Major Incidents	Disasters	Catastrophes
Impact	Very localized	Generally localized	Widespread and severe	Extremely large
Response	Local efforts	Some mutual assistance	Intergovernmental response	Major international response
Plans and procedures	Standard operating procedures	Emergency plans activated	Emergency plans fully activated	Plans potentially overwhelmed
Resource	Local resources	Some outside assistance	Interregional transfer of resources	Local resources overwhelmed
Public involvement	Very little involvement	Mainly not involved	Very involved	Extensively involved
Recovery	Very few challenges	Few challenges	Major challenges	Massive challenges

#### Proposed order of terminology for severity spectrum

Integrating descriptive words into an emergency management system improves mutual understanding and is easier to manage with minimal confusion. For instance, the terms “emergency,” “disaster,” and “catastrophe” have different levels of seriousness, where seriousness increases from emergency to disaster to catastrophe; therefore, these words should be used instead of the headings that merely state type 1, 2, 3 as IMTs. This change improves understanding at all levels and avoids confusion about whether type 1 or type 3 is the most critical. Naming the different categories and using plain language to describe the magnitude of a disaster allow for easier management at all levels. However, selecting the appropriate terms for different severity levels should be conducted with careful evaluation.

As a solution to the aforementioned inconsistencies, a standard terminology is required to describe the severity levels of natural disasters qualitatively because the descriptive terms are subjective. The terms “emergency,” “disaster,” and “catastrophe,” in this order, reflect an increasing order of seriousness of an event. However, 3 levels are not enough to clearly differentiate the impacts of disasters. Consequently, more levels are added: “apocalypse,” “calamity,” and “cataclysm.” However, the terms “apocalypse,” “calamity,” and “cataclysm” are typically colloquial, and they are not heavily used to describe disasters; hence, it is conjectured that people may randomly guess the level of seriousness that these words imply. Therefore, clear order of seriousness for the standard terminology is required to encourage a change in peoples’ response to disasters, and how they think about the severity of a disaster.

The proposed ranking of the selected terms in increasing order of severity is as follows: “emergency,” “disaster,” “calamity,” “catastrophe,” “cataclysm,” and “apocalypse.” The order of these terms is arranged considering the widely accepted understanding of the terms and their dictionary definitions. More details about the proposed order of terminology can be found in Analysis and classification of natural disasters (Caldera 2017). Nevertheless, since “apocalypse” has a religious connotation, it is replaced by “partial or full extinction.” This new term represents the most serious level.

#### Associated color code for the proposed terminology

According to the color coding system introduced in Step 2, the colors are assigned to each term as follows:

- Emergency is blue
- Disaster is green
- Calamity is yellow
- Catastrophe is red
- Cataclysm is purple
- Partial or full extinction is black

#### Proposed definitions of terminologies for the severity spectrum

The definitions for the terms are proposed in Table 7, and they are based on dictionary definitions and common usage; using any combination of the 6 terms to describe another is carefully avoided. The increasing order of seriousness is indicated by the terms’ definitions and the following methods of designation. The terms are listed from the lowest to highest order of seriousness:

- To describe circumstance (blue colored text), we use “event,” “disturbance,” and “upheaval,” which are modified by the adjectival forms “sudden,” “major,” “large-scale,” “very large-scale,” “extremely large-scale,” and “world-scale.”
- To describe impact (purple colored text), we use “damage,” “destruction,” and “devastation,” which are modified by the adjectival forms “significant,” “severe,” “widespread continental,” “global,” and “universal.”
- To describe injuries (green colored text), we use “many serious,” “major,” “massive,” and “uncountable.”
- To describe fatalities (red colored text), we use “some,” “many,” “great,” “extensive,” “unimaginable,” and “partial or full extinction.”

**Table 7:**
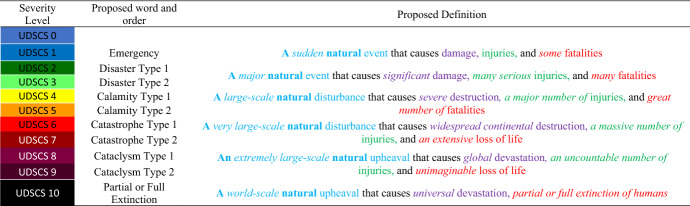
Qualitative Universal Disaster Severity Classification

Unlike the existing definitions, the proposed definitions provide a consistent method of differentiating as these definitions clearly articulate the real magnitude of different severity levels (Column 3 Table 7).

#### Combining the qualitative measure and the foundation of the UDSCS

The six terms “emergency,” “disaster,” “calamity,” “catastrophe,” “cataclysm,” and “partial or full extinction” are clearly defined to represent the severity levels of a disaster and the order of seriousness of the terms; however, there are 10 levels in the foundation of UDSCS that represent the vast range of impacts on both human and material damage that must be represented using these 6 words. UDSCS 0 is not considered here because it represents nondestructive events. The methodology combines qualitative measures (i.e., terminology used in the severity spectrum) and the foundation of the UDSCS (i.e., 10 levels and associated colors). The first destructive level of the UDSCS, UDSCS 1, is “Emergency” to indicate the impact of the disturbance on inhabited areas. The term “Partial or Full extinction” represents the last level of the UDSCS, UDSCS 10, and indicates total or partial destruction of the Earth. The levels in between are equally distributed among the remaining 4 words; each term has been subdivided into Types 1 and 2 of “Disaster,” “Calamity,” “Catastrophe,” and “Cataclysm,” as shown in Column 2 in Table 7. Thus, each severity level has unique words to describe it with clear definitions. These clearly defined terms help to quantify disaster events, to make comparisons, and to rank natural disasters more accurately. Even though the definitions are in English, they can be translated into most languages. Having clear definitions and a clear order of seriousness allows for easier recognition of an event occurrence and provides an overall picture of the severity of disasters to help emergency response management systems.

## Clear boundaries for the initial quantitative scale and UDSCS

### Step 4: Developing quantitative measure

Identifying relationships between factors that reflect the severity of an event aids in deciding what factors should be included in the multidimensional scale. Then, the most influential factors of severity are selected to develop the scale. Different methods can be used to identify the direct relationship between the factors. Statistical correlation can be used to identify the degree of linear relationship, and regression analysis can be used to identify the specific relationship. Different types of regression analyses and correlation methods are employed according to the type of variables used in the following analysis.

#### Identifying the most important influential factors related to severity

The most influential impact factors that can be considered for a multidimensional scale are from the socioeconomic factors listed in Step 1. Therefore, the impact of disasters on people, facilities, and the economy should be studied in detail to understand the severity of a natural disaster. Due to the lack of a complete recording system, the only socioeconomic factors considered in this correlation analysis are fatalities, injuries, missing persons, houses damaged/destroyed, and cost of damage in USD, as given in the NOAA database (National 2013a, 2013b). The Pearson correlation coefficient (ρ) is a common measure of association among continuous variables in tornado impacts, and Spearman's rho correlation coefficient (ρ′) obtains the relationship between ordinal interval variables in the effects of volcanic eruptions. Tables 8 and 9 show that all variables are positively correlated with ρ, ρ′ ≥ 0.5.

**Table 8:** Pearson correlation coefficient (ρ) for tornado effect factors (Caldera et al. 2018)

Variable	Injuries	Damage
Fatalities	0.781	0.829
Injuries		0.845

**Table 9:** Spearman’s rho correlation coefficient (ρ′) for volcanic effect factors (Caldera and Wirasinghe 2014)

Variable	Missing	Injuries	Damage Million USD	Houses Damaged
Fatalities	0.90	0.71	0.54	0.50
Missing		0.92	0.50	1.00
Injuries			0.64	0.54
Damage Million USD				0.90

Impact factors for tornado effects show a strong linear dependency as ρ is greater than 0.75, which normally means that, when one factor increases, the other factor is expected to increase. For example, an increase in the number of fatalities predicts an increased number of injuries and damage. However, when there are advanced warnings and mitigation measures, the number of fatalities and injuries can be minimized even if property damage increases. For the considered dataset, a natural linear relationship between these factors is investigated using multiple regression analysis and shown in Eq. (1). Table 10 shows that all coefficients in Eq. 1 for tornadoes are statistically significant because their p-values of 0.000 are less than 0.05.

1 $${\text{Fatalities}} = 1.26 + 0.03 * {\text{Injuries}} + 3.06 * 10^{ - 8} * {\text{ Damage}} $$

**Table 10:** Regression values for relationships between fatality and injuries, damage from tornadoes

Term	Coefficients	Std. Error	T value	P Value
Constant	1.26	.213	5.885	.000
Injuries	.03	.005	6.187	.000
Damage	3.06*10^–8^	.000	13.094	.000

The model that describes the relationships between fatalities, injuries, and damage fits 71% of the human impacts in data on tornadoes because the adjusted R-squared value is 0.71, which indicates a strong linear relationship. This R-squared value indicates that 71% of the variance in fatalities can be predicted from injuries and damage. Therefore, analyzing one factor can determine another using their linear dependency. For tornadoes, according to Eq. 1, the estimates reveal that for every additional 100 injuries, it is predicted that fatalities increase by 3, holding all other variables constant, and for every additional USD 100 million in damage, it is predicted that fatalities increase by 3, holding all other variables constant. Similarly, Caldera et al. (2016a) showed the linear relationship between impact factors and each type of disaster by analyzing tornadoes (Caldera et al. 2018), earthquakes (Esfeh et al. 2016), tsunamis (Caldera et al. 2016b), and volcanic eruptions (Caldera and Wirasinghe 2014).

Given that the impact factors are correlated (*ρ* ≥ 0.5) with each other, two approaches can be applied: measure the severity using one of these factors or develop a complex disutility function that includes several factors. Initially, the simplest approach of using one factor is selected to measure severity. The complex disutility function approach can be used to develop a multidimensional UDSCS. Therefore, 1 of the 5 factors (fatalities, injuries, missing persons, cost of damage, and houses damaged) is selected for the initial scale.

The number of fatalities is chosen as the most significant impact factor that represents the severity of all types of disasters because of these factors fatalities are the most serious factor and easy to define because of the finality of death. On the other hand, houses damaged closely relates to location, time, material, and size; cost of damage tends to increase with time because of inflation and wealth of the affected society; missing persons are eventually presumed dead and added to fatalities; and injuries are ambiguously defined because they can range from “small” to “moderate” to “severe” and may or may not include illness. In addition, populations are most sensitive to disastrous events with high fatalities, and many authors consider fatality a good measure of severity (de Boer 1990; Eshghi and Larson 2008; Gad-el-Hak 2008a; Löw and Wirtz 2010; Rodríguez et al. 2011; MunichRE 2013; Durage 2014; Hasani et al. 2014; Esfeh 2016; Yew et al. 2019). In addition, the number of fatalities is correlated with several factors; it highly correlates with injuries and missing persons and moderately correlates with houses damaged and cost of damage. Therefore, the number of fatalities is used to differentiate the levels of the initial severity scale. Extreme events based on fatalities are further analyzed using the extreme value theory.

#### Analysis of parent distribution of disaster events based on the most important influential factor

To understand the disaster continuum, a global-level dataset with different types of natural events must be considered. Therefore, 62 different types of disasters, such as global disasters (e.g., droughts, earthquakes, tsunamis, cyclones, and volcanoes), regional disasters (e.g., blizzards, general (river) floods, heat waves, tornadoes, and viral infectious diseases), and local disasters (e.g., avalanches, hailstorms, flash floods, forest fires, and landslides) are included in this analysis with the frequency distribution shown in Table 11. The data in the EM-DAT global loss database for all types of natural disasters from 1977 to 2013 inclusive are considered. Although data from 1900 to 2013 were available in the EM-DAT database, CRED restricts the maximum amount of data issued to around 10,000 records, and since the recording system improved after 1980, more recent historical records were chosen (Centre 2013).

**Table 11:** Event distributions according to their groups and main types of disaster profile

Group	Events %	Main Type	Category	No. of Events
Biological	12	Animal stampede	Animal stampede	0
		Epidemic	Bacterial infectious diseases	646
			Parasitic infectious diseases	42
			Viral infectious diseases	394
			Other epidemics	137
		Insect infestation	Grasshopper/Locust/Worm	78
Climatological	12.1	Drought	Drought	506
		Extreme temperature	Cold wave	260
			Extreme winter condition	59
			Heat wave, Icing, Freezing rain	141
		Wildfire	Forest fire	246
			Scrub/Grassland fire, Bush/Brush fire	76
			Other wildfires	15
			Land fire	0
Extraterrestrial	0	Meteorite/Asteroid	Meteorite/Asteroid	0
Geophysical	9.9	Earthquake (seismic activity)	Ground shaking	838
			Tsunami, Other seismic activity	27
		MMD	Landslide-MMD	25
			Other MMD, Debris flow, Sudden subsidence, Mudslide, Snow avalanche, Rock fall, Avalanche	18
		Volcano	Volcanic eruption	160
Hydrological	39.3	Flood	Flash flood	481
			General flood	2368
			Storm surge/coastal flood	76
			Other flood, General flood/Mudslide	815
		MMW	Landslide-MMW	432
			Other MMW, Debris flow, Sudden subsidence, Mudslide, Snow avalanche, Rock fall, Avalanche	79
Meteorological	26.7	Storm	Extratropical cyclone	99
			Hailstorm	93
			Severe storm	136
			Snowstorm	73
			Snowstorm/Blizzard	49
			Blizzard, Blizzard/Tornado, Blizzard/Dust storm, Dust storm, Sandstorm/Dust storm, Sandstorm, Snowstorm/Sandstorm, Extratropical cyclone (winter storm), Severe storm/ Hailstorm	25
			Thunderstorm	87
			Tornado	221
			Other local/ Convectional storm	22
			Tropical cyclone	1437
			Other storm	646

MMD = Mass Movement Dry; and MMW = Mass Movement Wet

This analysis consists of 5 out of 6 main groups of natural disasters, and the frequency distributions of these 37 different categories of disasters are shown in Table 11. The considered dataset includes 59 secondary sub-types of disasters (Wirasinghe et al. 2013a), but it does not include data on meteorites/asteroids in extraterrestrial events, animal stampedes in biological events, nor land fires in climatological events. Another drawback of the considered dataset is the database records are grouped by country. For example, 2004 Indian Ocean tsunami data were distributed over 12 different records according to the 12 affected nations. Therefore, the actual impact of some large events is not properly captured.

It is essential to determine the statistical characteristics of fatalities and the best probability distribution fit that are able to describe fatalities. There are 10,805 records of fatalities out of 10,807 records from 1977 to 2013 logged in the EM-DAT database, with minimum 0 and maximum 300,000. The mean of fatalities is 258.04, and the standard deviation is 5491.18 with 38.65 skewness and 1,686.98 kurtosis, which means the parent probability distribution of fatalities has more extreme events at longer fatality numbers, i.e., a long right tail.

**Fig. 1:**
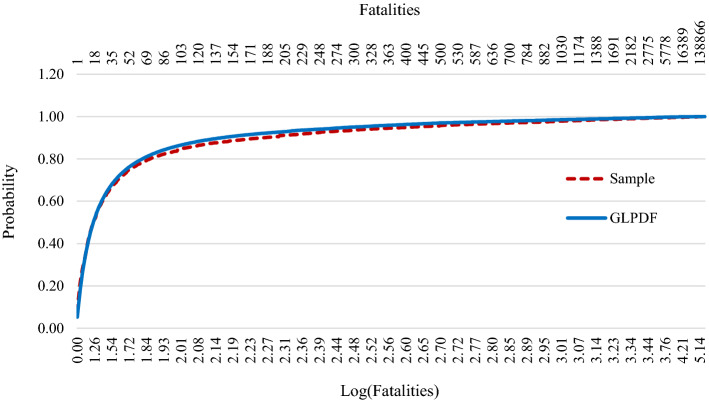
Cumulative sample distribution of fatalities in a natural logarithm scale with an approximate GLPDF

Determining the distribution that historical data follow is necessary to estimate the probability of future events for a given number of fatalities. To better fit the distribution, fatalities are transformed into natural logarithms after eliminating zeros (3287 records) and no records of fatality (2 records) from the 10,807 records (Caldera 2017). The logarithmic data of fatalities of 7518 records have a mean of 1.275 and a standard deviation of 0.769 with 0.610 skewness and 1.054 kurtosis, with minimum 0 and maximum 5.4771. Close approximate distributions were fitted to the logarithmic data of fatalities. The probability density function (PDF) of the generalized logistic distribution (GLPDF) with μ equals 1.224 and σ equals 0.424, which is an approximate parent distribution fit for fatalities, as shown in Eq. (2). The cumulative distribution function (CDF) of the fitted GLPDF and the sample CDF is shown in Fig. 1.

2 $$f\left( x \right) = \frac{{e^{{ - \left( {\frac{x - \mu }{\sigma }} \right)}} }}{{\sigma \left[ {1 + e^{{ - \left( {\frac{x - \mu }{\sigma }} \right)}} } \right]^{2 } }}; \quad {\text{where }}\sigma > 0,{\text{ and }}\;0{ } < x < + \infty$$

#### Method for identifying extreme disasters to represent the severity spectrum

Extreme value theory can be used to study the behaviors and destructive capacity of strong, violent, infrequent, disasters. Extremes are low probability events, which are located on the tail of the parent PDF. In this case, the right tail of the parent PDF is considered because the extremes are largest or maxima of severe events. These extreme events are selected to fit an extreme value probability distribution function (EPDF). The EPDFs are limiting distributions and essential to evaluate the probability of extreme disasters. There are three models available, block maxima, R^th^ order statistic, and peak over threshold, to identify the extreme events (Kotz and Nadarajah 2000; Coles 2001; Reiss and Thomas 2007). The peak over threshold model only contains high extreme records because it is bounded below; consequently, a full range of extreme disaster types with fatalities is not included. To select the extreme fatalities using block maxima or R^th^ order method for all types of natural disasters, each block is considered as a different type of natural disaster; otherwise, the method will be biased to large-scale disasters, and it will not select fatalities from small-scale disasters when small-scale and large-scale disasters are grouped together. The number of extremes gradually increases according to the order statistics as shown in Eq. (3) (Caldera 2017). Because R varies from 1 (i.e., block maxima) to R, R different extreme fatality value datasets representing all types of natural disasters are selected for the analysis.

3 $${\text{Sample size of}}\;R^{th} {\text{order extreme dataset }} = {\text{ Number of categories}}\; * R^{th} {\text{order}}$$

**Fig. 2:**
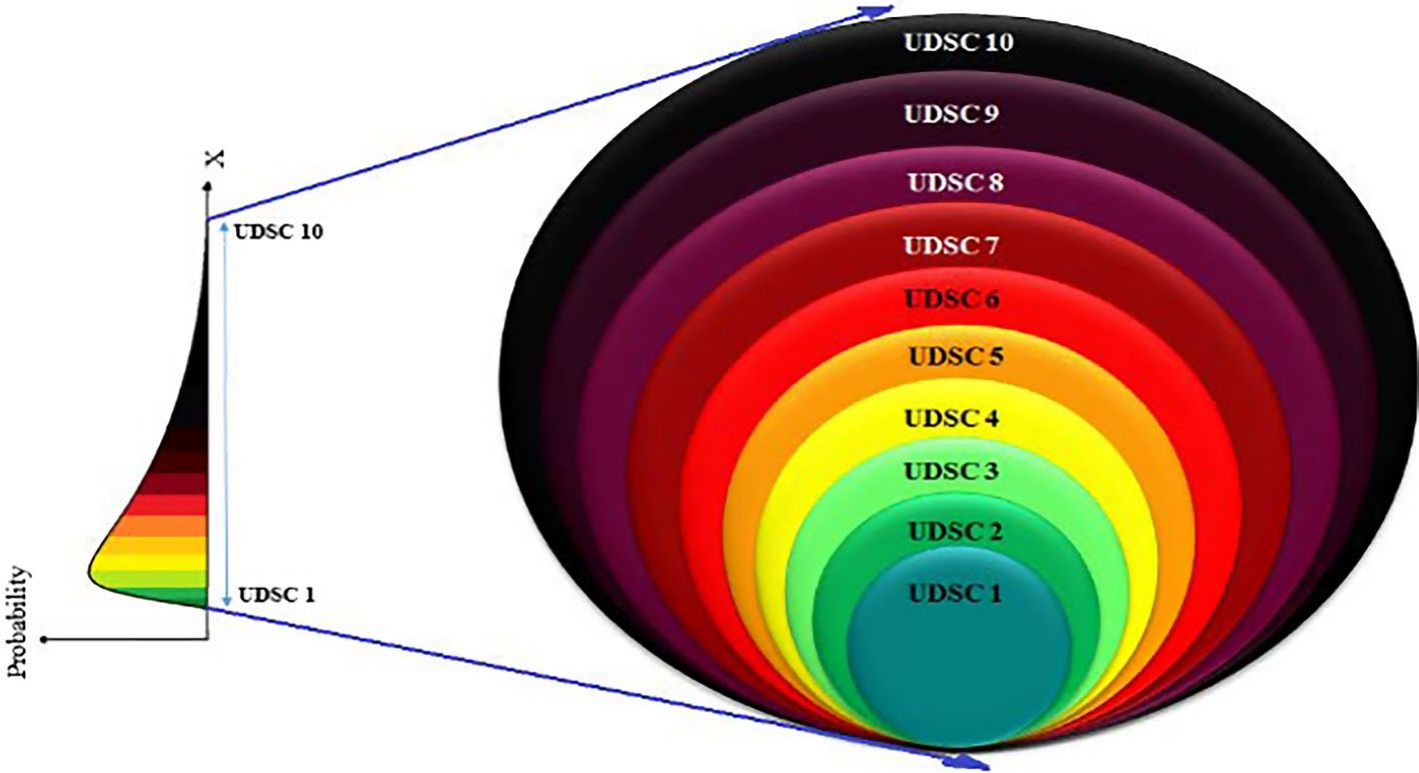
Probability distribution and severity levels

Therefore, substantially fewer numbers of extreme records are included in the block maxima model compared to the R^th^ order statistic model. Therefore, of the three methods, R^th^ order statistic was used in this analysis because it selects a considerable number of extremes for each type of disaster and covers the full range of severity (i.e., fatalities) ranging from small-scale to large-scale disasters. Extremes in the R^th^ order statistical model are distributed as a generalized extreme value distribution (GED). GED can be further explained by either Gumbel (GE0), Frechet (GE1), or Weibull (GE2) distributions (Kotz and Nadarajah 2000; Coles 2001; Reiss and Thomas 2007). Different types of EPDF are fitted for the R^th^ order statistical models. The best fitted EPDF of extremes is used to define the ranges of severity levels as shown in Fig. 2.

#### Analysis of extreme disaster events based on the most important influential factor

To apply the extreme value theory to the random variable (number of fatalities), each type of natural disaster represents one block in the extreme value analysis. Although there are 59 different secondary sub-types of disasters recorded in the EM-DAT database from 1977 to 2013, the same categorizations (blocks) cannot be used to extract the extreme values as some secondary sub-types have none or only one, two, or less than 10 recorded events (e.g., blizzards, dust storm, freezing rain, icing, sandstorm, and snow avalanche). In these cases, there are not enough extreme values to represent the highest R^th^ order statistic. Therefore, a new categorization is introduced that combines the categories that had a smaller number of events (e.g., parasitic infectious diseases, extreme winter conditions, and other wildfires). To reflect a reasonable number of data points in each block, first, the different types of disasters are grouped according to their secondary sub-type, sub-type, or main type if there are not enough events in the category to represent the R^th^ order; then, fatalities are ordered from highest to lowest for each category. Subsequently, the first R number of fatalities in each block is selected. Therefore, the following categories are combined:

- “Parasitic infectious diseases” and “Other epidemics” are combined into “Other epidemics”;
- “Cold wave” and “Extreme winter conditions” are combined into “Cold wave or Extreme winter conditions”;
- “Scrub/Grassland fire,” “Bush/Brush fire,” and “Other wildfires” are combined into “Other wildfires”;
- “Tsunami,” “Other seismic activity,” “Mass movement dry landslide,” “Other mass movement dry,” “Debris flow,” “Sudden subsidence,” “Mudslide,” “Snow avalanche,” “Rock fall,” and “Avalanche” are combined into “Other geophysical events”; and
- “Other Local/Convectional storm,” “Snowstorm/Blizzard,” “Blizzard,” “Blizzard/Tornado,” “Blizzard/Dust storm,” “Dust storm,” “Sandstorm/Dust storm,” “Sandstorm,” “Snowstorm,” “Sandstorm,” “Extratropical cyclone (winter storm),” and “Severe storm/ Hailstorm” are combined into “Other local/Convectional storm.”

Consequently, there are 27 categories and each category corresponds to one block, and their 5^th^ order, 10^th^ order, 15^th^ order, …, up to 70^th^ order statistics (i.e., 14 different extreme datasets) are analyzed. The sample sizes of these 14 extreme datasets of R^th^ order statistics increase by multiples of 27 according to Eq. (3) because this analysis considers 27 different categories of natural disasters (27 blocks).

**Fig. 3:**
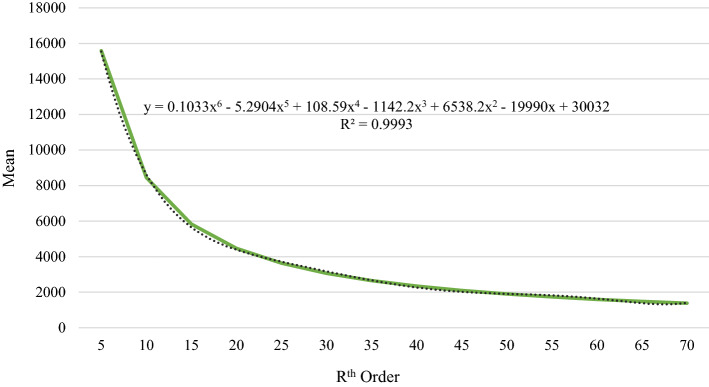
Mean distribution of R^th^ order extremes

The distribution of the mean (and its trend line) of these 14 extreme datasets are shown in Fig. 3. The trend lines of the mean are significantly close to the actual values because the R-squared value is close to 1 (R^2^ > 0.99). The first derivative of these fitted trend lines measures the rate of change of the mean, while the second derivative measures whether this rate of change is increasing or decreasing. The mean value stabilizes when R^th^ order increases because the rate of change is decreasing when R^th^ order increases. Therefore, the mean slowly decreases and converges to its full sample value (i.e., 258.04) when R^th^ order increases.

According to the extreme value distribution selection procedure, the 70^th^ order statistic is considered as the best minimum R^th^ order statistic to estimate the probabilities of severity levels of fatalities (Caldera 2017). Then, the extreme value distributions (GE1, GE2, and GED) are fitted to 70^th^ order statistic to assess the extreme natural events. A wide range of fatalities, 0 to 7.674 billion (the world’s population, World 2019a), can be concentrated into 10 levels using the log scale. Therefore, the magnitudes of the severity level boundaries are defined based on the logarithm of the fatalities.

#### Combining the initial quantitative measure (i.e., the proposed ranges of the severity spectrum) and the foundation of the UDSCS

The estimated probabilities and the sample probabilities of severity levels 0 to 10 according to the foundation of UDSCS from Step 2 are shown in Table 12. The full sample dataset from 1977 to 2013 and the 70^th^ order statistic extreme dataset are used to calculate sample probabilities for severity levels. The 70^th^ order statistic sample for extreme events represents 17.49% of the full dataset. The estimated probabilities of severity levels in Table 12 are calculated using the fitted 70^th^ order Frechet (GE1), Weibull (GE2), and generalized extreme value distribution (GED).

**Table 12:**
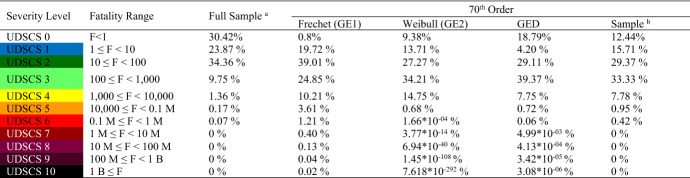
Estimated probabilities of severity levels

M = Million; B = Billion

^a^Sample of historical data from 1977 to 2013 (10,805 events out of 10,807 records)

^b^Sample of extremes in the 70^th^ order statistic (1890 events out of 10,807 records)

Out of the 10,807 sample events, two events did not have fatality records. Thus, considering the remaining 10,805 sample events (Column 3 Table 12), only 69.58% of the full dataset had at least one fatality, while 30.42% of events recorded zero fatalities. In addition, 12.44% of extreme events of the 70^th^ order sample (Column 7 Table 12) recorded zero fatalities, which means that the 70^th^ order statistic of extreme datasets consists of the full range of extremes from small-scale to large-scale disasters. Moreover, GE1, GE2, and the GED estimates 0.80%, 9.38%, and 18.79% probabilities for zero fatalities, respectively.

**Fig. 4:**
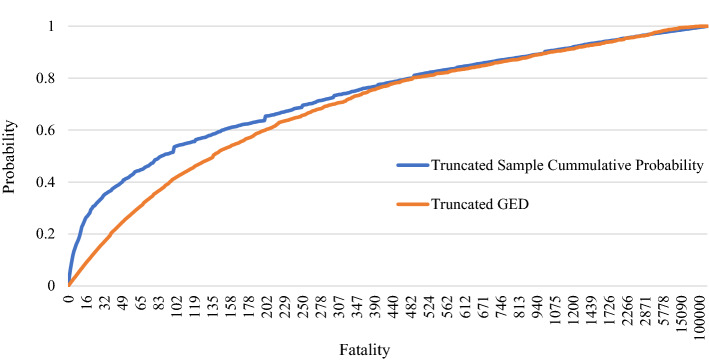
Cumulative sample distribution of fatalities with an approximate 70^th^ order GED

Compared to the estimated probabilities of GE1, GE2, and GED, only the 70^th^ order sample probabilities for levels 0, 1 and 3 are closer to GE2 than GED; all other severity levels of the 70^th^ order sample probabilities are closer to GED than GE2 or GE1. Additionally, GE2 gives significantly lower probabilities compared to GE1 and GED for the estimated probabilities of higher severity levels (from level 6 to level 10) although the 70^th^ order sample has 0.42% representation for UDSCS 6 or higher events. In contrast, GE1 yields significantly higher probabilities compared to GE2 and GED for the estimated probabilities of levels 7 to 10. For example, 2 out of 10,000 severe natural disasters can be considered as severity level 10 events (i.e., fatalities exceed 1 billion) according to the fitted GE1, which is a higher probability for partial or full extinction. However, compared to the estimated probabilities of GE1 and GE2, the estimated probabilities of GED are closer (and more reliable) to the 70^th^ order sample probabilities for higher severity levels. Table 12 illustrates that 5 out of 100,000 severe natural disasters will have 1 million to 10 million fatalities, 4 out of 1 million will have 10 million to 100 million fatalities, 3 out of 10 million will have 100 million to 1 billion fatalities, and 3 out of 100 million will have more than or equal to 1 billion fatalities, according to the fitted GED. Thus, the fitted GED of 70^th^ order statistic is suitable to calculate the approximate probability values of natural disaster severity levels (Column 6 Table 12). The CDF of the fitted 70^th^ order GED as shown in Eq. 4 and the sample CDF are shown in Fig. 4. Note that the probabilities of Fig. 4 are truncated because the cumulative probability value of zero fatalities is 0.18 and the sample probability of zero fatalities is 0.12.

4 $$F\left( x \right) = e^{{ - \left[ {1 + \gamma \left( {\frac{{x_{i} - \mu }}{\sigma }} \right)} \right]^{{\frac{ - 1}{\gamma }}} }} { }; \quad {\text{where }}\mu = 44.396; \quad { }\sigma = { }106.060; \quad { }\gamma = { }0.924$$

### Step 5: Combining quantitative and qualitative measures

As a way to measure the severity of natural disasters, an UDSCS is developed that has 0–10 levels and is designed by combining both quantitative (initial) and qualitative measures to differentiate each level as shown in Table 13. Each severity level has a fatality range, expected probability for the level, and a color code. In addition, each severity level has a unique word to describe it and is clearly defined. Examples are also provided for each level and are drawn from historical events. For example, UDSCS 1, “Emergency,” accounts for situations that have between 1 and 10 fatalities, and UDSCS 10, “Partial or Full Extinction,” is defined as situations that exceed one billion fatalities.

**Table 13:**
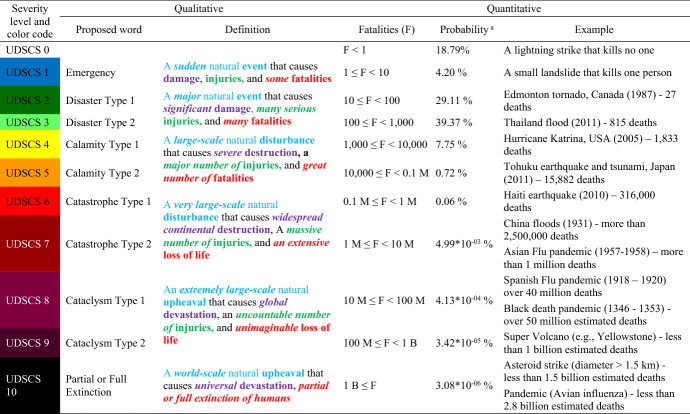
Initial Universal Disaster Severity Classification—Fatality based

M = Million; B = Billion

^a^Estimated approximate probabilities according to the fitted GED of 70^th^ order statistic

Almost everything in this table is novel. For the first time, a 0–10 level ranking is proposed, which make sense because it is a log scale that can cover wide ranges in terms of socioeconomic factors. Although each severity level increases by a power of 10, the probability of events that fall within the higher ranges of the scale is small. The probability of a very high classification is low for severe natural disasters as these events are rare. Furthermore, the base 10 measurement is easy to remember and meaningful because it clearly differentiates one severity level from another.

The estimated probabilities of these levels are calculated using the approximate best-fitted 70^th^ order statistic GED (Column 4 and 5 Table 13). UDSCS level 6 or higher disasters are expected to have very small, estimated probabilities according to the fitted 70^th^ order GED. These probabilities are estimated using a low exact number of severe events (7 historical records for UDSCS 6 and higher events for the 70^th^ order sample or full sample dataset from 1977 to 2013) because there are no historical records for UDSCS 7 or higher disasters in the considered dataset; however, there is geographical evidence of natural disasters that have occurred in the past. Therefore, the estimated probabilities for last four levels (UDSCS 7 to 10) are very low, and these probabilities are indicative of their severity range.

According to the considered dataset, the maximum fatality record is 300,000, which falls into Catastrophe Type 1 (UDSCS 6). However, according to the fitted 70^th^ order GED, it is expected that 5 in 1 million severe events are Catastrophe Type 2 or higher disasters. The severity levels of the 2 worst extreme natural disasters that have occurred in history, and for which data are available, are the Black death pandemic that occurred between 1346 and 1353 where more than 50 million fatalities were recorded, and the Spanish Flu pandemic that occurred between 1918 and 1920 where more than 40 million fatalities were recorded (Saunders-Hastings and Krewski 2016); these events are categorized as Cataclysm Type I (UDSCS 8). Additionally, the Asian Flu pandemic that occurred between 1957 and 1958 and resulted in more than 1 million deaths (Rajagopal and Treanor 2007) and China’s 1931 flood that resulted in more than 2.5 million deaths are categorized as Catastrophe Type 2 (UDSCS 7). Therefore, the above estimates are reasonable considering events that are not included in the analysis. Furthermore, there can be disasters that are not recorded in the databases, such as extraterrestrial events or the combined impact of extreme disasters such as an earthquake and tsunami, that affected more than one country.

Disasters, such as meteoroid impacts, have the potential to vary from “Emergency” (UDSCS 1) to “Partial or Full Extinction” (UDSCS 10). Although there are no recorded fatalities caused by a meteoroid impact, the falling of meteoroids gained attention after the Russian meteor strike in 2013 that injured more than 1,000 people. Also, there are many studies about extinction risks, such as super-volcanic eruptions or major asteroid impacts. The studies estimated the number of deaths that might occur, but the probability of these events occurring is very low. According to the Planetary Society, an asteroid larger than 1 km across is big enough to threaten global destruction, and astronomers estimate such objects have a 1 in 50,000 chance of hitting Earth every 100 years (Kettley 2020a). These kinds of asteroid strikes can be categorized as Partial or Full Extinction (UDSCS 10). Scientists have modeled that a super-eruption might kill 10 percent of the global population (i.e., more than 700 million) (Walsh 2019), and therefore, super-eruptions can be categorized as Cataclysm Type 2 (UDSCS 9). However, Dr. Jerzy Żaba, a geologist, estimates the Yellowstone volcano could trigger global climate change, and about five billion might die from starvation in the aftermath of that eruption (Kettley 2020b), so the combined impact of the eruption and the aftermath may lead to Partial or Full Extinction (UDSCS 10).

Predictions from the model need to consider possibilities outside the estimated probabilities and must be used with caution because decision makers may believe them to be absolute. According to the estimated probabilities of 70^th^ order GED, 19 out of 100 extreme natural disasters are less than UDSCS 1. They can be disasters that are not recorded in the database (less than 10 fatalities) or zero fatality events, such as insect infestations and lightning strikes, according to the historical events recorded. Four out of 100 severe natural disasters can be considered as UDSCS 1 events that have 1 to 10 fatalities. An example of a UDSCS 1 is icing, which is any deposit or coating of ice on an object that can seriously hamper its function and is considered an extreme temperature condition grouped under climatological disasters. Note that the EM-DAT database records events that have less than 10 fatalities, if 100 or more people are reported as affected or there has been a call for international assistance/declaration of a state of emergency. Thus, the estimated probabilities of UDSCS 0 and 1 are also conditional to the above data entering criteria.

Severe natural disasters fall under Disaster Type 2 (UDSCS 3) according to the analysis; 39 out of 100 severe disasters will have 100 to 1,000 fatalities. According to historical events, bush and forest fires, cold waves, avalanches, snow avalanches, rock falls, storm surges/coastal floods, sudden subsidence, debris flows, mudslides, tornadoes, and storms (severe, hail, dust, and local) can be classified as UDSCS 3. The second most likely severity level that severe natural disasters can fall under is Disaster Type 1 (UDSCS 2); 29 out of 100 severe events will have 10 to 100 fatalities. According to the data, freezing rains, scrub/grassland fires, other wildfires, other seismic activity, and storms (snow, winter, sand, blizzard, thunderstorms, and extratropical cyclones) can be classified as UDSCS 2. Thus, 68% of severe natural disasters will have 10 to 1000 fatalities and fall under either Disaster Type 1 or Type 2 (UDSCS 2 or 3).

The next major natural disaster will have a 7.75% chance of causing between 1000 and 10,000 deaths. In other words, 775 out of 10,000 extreme disasters can be classified as UDSCS 4, Calamity Type 1. Most biological events, such as epidemics (e.g., parasitic and bacterial infectious diseases), extreme winter conditions, floods (general and other), landslides, and other storms fall under this category.

Extreme disasters, such as volcanoes, flash floods, and heat waves can be classified as UDSCS 5, which means 72 out of 10,000 severe natural disasters can be considered as Calamity Type 2 events that have 10,000 to 0.1 million fatalities. Earthquakes, tsunamis, tropical cyclones, and droughts have the ability to reach UDSCS 6, and 6 out of 10,000 severe disasters can be classified as Catastrophe Type 1 events.

This universal classification system compares the severity of different types of disasters and presents an overall picture of severity levels (Caldera 2017). According to this classification, local disasters cover the lower levels, whereas the disasters with potential regional- or global-level impacts cover the upper levels.

However, it should be noted that the extreme fatality analysis used historical events from 1977 to 2013 recorded in the EM-DAT database, and none of these records included events that had fatalities exceeding 300,000 (Catastrophe Type 1). In addition, as mentioned previously, the database records depend on the country (e.g., the 2004 Boxing Day tsunami data are not recorded as one event but 12 different events because 12 different nations were affected). Moreover, there are events before 1977 (e.g., the 1931 China flood, classified as Catastrophe Type 2) that this analysis does not cover, and there is the possibility that future events exceed 300,000 fatalities.

In addition, simultaneous disaster events (e.g., an earthquake and tsunami striking or the impact of a hurricane and peripheral tornadoes) are not considered in this analysis. These events can cause the classification level to increase by one or more levels.

Additionally, infrastructure failure can be added to an event or simultaneous events, for example, the nuclear plant failure subsequent to the Great North East Japan Earthquake and Tsunami. A meteoroid impact on land close to population centers or in the ocean (causing massive tsunamis) could cause millions of fatalities.

Although the analysis is subject to many limitations, it provides a good foundation to develop an advanced multidimensional scale to classify disaster occurrences worldwide based on a combination of several independent factors. This analysis also provides an overall picture of the severity of each type of disaster. This kind of scale makes it easy to recognize an event occurrence and enter it into a database.

## Natural disaster severity classification

Table 14 illustrates the levels covered by each disaster group and the breakdown of geophysical disasters according to the historical sample data in the EM-DAT database from 1977 to 2013. The covered severity levels are indicated using “√” (checkmark) and the respective severity color code is also given. Table 14 compares and contrasts the severities of the five main groups, biological, hydrological, meteorological, climatological, and geophysical, considered in the analysis. In addition, it presents an overall picture of the severity levels. As an example, the severity spectrum of geophysical disasters is shown in this table.

**Table 14:**
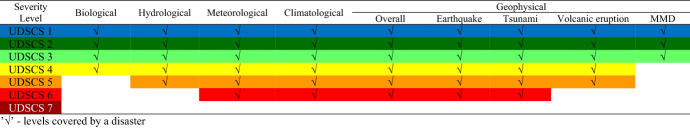
Natural Disaster Severity Classification

Moreover, the disaster classification of geophysical disasters is validated using this table. The maximum fatality record (China in 1556) for earthquakes in the NOAA database from 2000 B.C.E. to 2015 C.E. is 830,000 (Esfeh et al. 2016). For a tsunami, the most fatalities that occurred is 300,000 (India in 1737) as recorded in the NOAA database using records from 2150 B.C.E. to 2015 C.E. (Caldera et al. 2016). Therefore, earthquakes and tsunamis can reach severity level 6 in the UDSCS (100,000 ≤ Fatality < 1,000,000). For volcanic eruptions, as recorded in the NOAA database from 4360 B.C.E. to 2013 C.E., the maximum fatality record is 30,000 (El Salvador in 450) (Caldera and Wirasinghe 2014). Therefore, volcanic eruptions can reach level 5 (10,000 ≤ Fatality < 100,000). Consequently, geophysical disasters can go up to a severity level of 6 in the UDSCS. This conclusion is similar to the disaster classification conclusion that uses sample data from 1977 to 2013 (Table 14, columns 6 to 9). Although the disaster classification represented in Table 14 uses historical disasters from 1977 to 2013, the table covers the full range of disasters recorded in history for different geophysical disasters (i.e., tsunamis, earthquakes, and volcanic eruptions). Therefore, this comparison confirms that despite various limitations, the proposed disaster classification and the Universal Disaster Severity Classification Scheme are closer to the actual situation for many disasters.

## Global databases and research limitations

Historical records are the basis for understanding the severity of a disaster, and numerous techniques have been used to record historical events (National 2007). However, data collection standards vary among countries, and therefore, comparisons across space and time are difficult.

Comparing different events and obtaining a sense of scale are problematic due to deficiencies in databases. Some deficiencies in global databases are due to the following:

- Incomplete data: some databases do not record all the necessary information;
- Inaccurate data: global databases lack common standards;
- Missing data: some events are not entered in the dataset because of the definitions or requirements of that database.

Although the number of reported natural disasters is increasing, in general, records are incomplete. Historical reports contain some, but not all, important data; most contain only a brief and often ambiguous description (Newhall and Self 1982). In addition, current records can be inaccurate and ambiguous, which complicates the relationship between impact factors and the severity of a natural disaster. For example, the reported number of homeless people was zero in the Great East Japan (GEJ) earthquake in the EM-DAT international disasters database of the Centre for Research on the Epidemiology of Disasters (CRED) (Centre 2013). However, several thousand homes were washed away in the GEJ earthquake, leaving many people homeless. Temporary houses that were provided were in use 4 years after the event. The statistics in this example indicate that there are some concerns about information management, information processing, and how these variables are defined in global databases.

The lack of common terminology to identify the scale of a destructive event is an issue in information management and processing (Hristidis et al. 2010), which can lead to “…inconsistent reliability and poor interoperability of different disaster data compilation initiatives” (Below et al. 2009). It is not uncommon for numerous records to exist for the same event, sometimes with different numbers. For example, there are different fatality records from different sources for the 1815 volcanic eruption of Mount Tombora in Indonesia. “Victims from volcanic eruptions: A revised database” (Tanguy et al. 1998) recorded 11,000 fatalities due to the volcanic eruption (with an additional 49,000 fatalities associated with the eruption but caused by post-eruption famine and epidemic disease). However, the National Oceanic and Atmospheric Administration (NOAA) database recorded 10,000 fatalities from the eruption (with 117,000 total fatalities in the aftermath of the eruption) (National 2013a). Given that one can count direct fatalities or fatalities in the aftermath (e.g., secondary disasters, such as climate anomalies, altered weather patterns, ground deformation, ash fall, pollution, starvation, landslides, and tsunamis), this adds to the possibility of inaccuracies in the databases. Several such discrepancies exist among various sources, and they complicate the interpretation of trends in disaster data.

Moreover, one disaster may lead to another disaster, which results in conjoint disaster records, and therefore, separating the impacts can be problematic. Thus, the nature of a disaster, whether it is primary or secondary, is one of the main issues in distinguishing one disaster from another (Wirasinghe et al. 2013a). Additionally, databases that compile disaster events at the national level face issues with disasters that have impacts at the regional or continental level. The same disaster event can also impact countries differently (Löw and Wirtz 2010), and thus, the interpretation of scale of a disaster can be different from one country to the other (Wirasinghe et al. 2013b).

Further, different databases have different criteria for including a disaster in their databases. For example, in the EM-DAT database, a disaster has to result in: 10 or more people have been reported as killed and/or 100 or more people are reported as affected and/or there has been a call for international assistance or a declaration of a state of emergency. In contrast, events that are entered in the Munich RE global loss database, NatCatSERVICE, are those that have resulted in human or material loss (MunichRE 2013). Thus, a given event occurrence recognized as a disaster and logged in one database may not be recorded in another. Events such as those with less than 10 fatalities, with less than 100 people affected, and with a monetary impact, but not declared as a state of emergency, are archived in NatCatSERVICE but not in EM-DAT. Therefore, databases that use different entry criteria may give different interpretations for the same event (Below et al. 2009).

A lack of data and incomplete/inaccurate data can prevent in-depth analysis. Although historical inaccuracies in past records are unavoidable, going forward, inaccuracies should be avoided, if possible. To have accurate records, we need the following:

- Improved data (i.e., universal, complete, comprehensive, unambiguous, and accurate).
- Enhanced databases (e.g., record and retrieve joint or separate data and global disasters or the subdivisions of a continental, regional, or national records)
- Improved information management and processing (e.g., data collection and entry criteria standards).
- Precise disaster terminology (e.g., standardized terms for easily recognizing an event occurrence).

However, these requirements do not stand alone but are interconnected with each other. Hence, consistent interpretation, a proper scale, good understanding of each disaster, and an expanded recording system are required to accomplish this goal. Therefore, a global disaster classification system is an important contribution to improving the quality and reliability of international disaster databases (Löw and Wirtz 2010).

## Significance of the UDSCS

### Common severity scale for all types of natural disasters

The main advantage of this new UDSCS is that it will provide a common platform to compare natural disasters. Therefore, comparisons across regions and time for any type of natural disaster is feasible using this novel universal classification system. This knowledge can be used for impact assessments for different hazards (see Sub-Sect. 2.4).

In addition, this universal system is not confined to disasters resulting from rapid onset, relatively clearly defined events such as earthquakes, tsunamis, and tornadoes. Disasters resulting from events that are more diffuse in space and time are also incorporated, such as droughts, famine, pollution, and epidemics. Conditions that become disastrous, but with less clear start and end points, are also incorporated because the UDSCS also considers slow moving disasters.

As this universal system considers the world’s population, it incorporates conditions that become extinction events or massive phenomena, such as a major asteroid strike, super-volcanoes, or a meteoroid impact. Analyzing the risks and responses to events that have the potential to cause the full or partial extinction of the human race is crucial but curtailed as obviously there are no historical records, but there are geographical records.

Another advantage of this universal classification system is that it is expected to generate a consistent standardized communication platform to describe the impact of disasters for all stakeholder groups, such as civilians, responders, and policy makers. The initial UDSCS also provides a foundation to develop an advanced scale to classify and compare disaster occurrences worldwide, but the analysis is subject to many limitations. In addition, the UDSCS will improve disaster terminology. Furthermore, in response Löw and Wirtz (2010) comment about a global disaster classification (see Sect. 7), the UDSCS will improve the quality of data, recording systems, and databases by providing precise disaster terminologies. Most importantly, the proposed UDSCS will improve communication and understanding of disaster risks, which aligns with the priority of the Sendai Framework for Disaster Risk Reduction 2015–2030 (United 2015).

### Improved understanding of disaster risk

The UDSCS is not a replacement for estimate firsthand damage, but the universal system can support prioritization during the early stages of a response. As the response to a disaster continues, the UDSCS can be updated to consider improvements to the severity scale and sources of data (quality, timeliness, and scale) that are validated via firsthand reports and changing requirements. Therefore, this new universal severity classification system is expected to provide benefits to several groups:

1. Emergency responders and disaster managers
2. National/regional/local governments
3. Relief agencies and NGOs
4. Reporters and media
5. General public
6. Insurance managers and estimators
7. Database/information managers
8. Research community

#### Emergency response and disaster management

Disaster managers and emergency respondent personnel can gain a clear sense of scale of the severity of each type of disaster by considering the expected probabilities according to historical disasters. Also, they can have an overall picture of a disaster because UDSCS provides relative comparisons among disasters of various degrees and ranks natural disasters using a set of criteria. This knowledge can be used to deploy resources as needed when disaster strikes, and it can be used for pre-planning (see Sub-Sect. 2.4).

The initial assessment of a disaster is based on estimates made shortly after the event strikes, and it is frequently updated. For example, first evaluations are used for initial planning, such as whether to call a state of emergency, evacuate, request international assistance, or involve military forces. Other decisions regarding planning include the following: resources, such as food, water, medicine, sanitation, and clothes, that should be stored and delivered to the stricken area; hospitals that should be assembled and to what extent; and shelters to mobilize, where to set up temporary housing, and for how long. By having an overall picture of the severity of disasters, emergency response management organizations, disaster managers, first responders, government stakeholders, relief agencies, and NGOs can rapidly estimate the potential impact of a natural disaster, and then, they can quickly respond by properly allocating the appropriate resources, expediting mitigation, and accelerating the recovery processes (Caldera et al. 2018), which cannot be done using the current scales.

No matter the type of disaster, similar resources are managed by personnel who allocate available emergency vehicles, essential resources, temporary hospitals, temporary housing, etc. Mitigation efforts are dependent on the estimated disaster impact. Identifying the disaster impact properly, and in a timely manner, is crucial because lives depend on these decisions. Inconsistent identification of disaster impacts mean that disaster managers may either over- or undercompensate in their allocation of resources for mitigation. Overcompensation could result in a large waste of resources, while undercompensation could increase the severity of an impact. In addition, one city can have different types of disasters, but the same personnel respond to these events.

Moreover, populations are most sensitive to disasters that have high human impacts. Therefore, a severity scale based on human impacts should be used for preparedness and mitigation methods; warnings, evacuation, public awareness, disaster education, and disaster drills can help change public opinion regarding the impact of disasters; may gain the public’s attention and increase trust in the techniques used by emergency management systems and emergency responders. Thus, response time to warnings can be decreased, and response rates can be increased if the proposed terms are used. Consequently, public awareness, education level, and response rate to warnings can be increased using the UDSCS because a direct relationship between a disaster and the probability of human impact are made explicit. As Durage (2014) indicated, “The frequent occurrence and high intensity of natural disasters can impose irreversible negative effects on people. Taking mitigation actions well in advance can avoid or significantly reduce the impacts of disasters.” Although it is difficult to avoid property damage due to the sudden onset of a natural disaster, if proper classifications and terminology are used in an emergency management system, fatalities and injuries could be minimized by taking appropriate actions, such as issuing warnings on time and raising public awareness. Therefore, warnings indicating the severity of a natural disaster can be communicated using the clearly defined terms in the UDSCS, and meaningful communication regarding life-threatening situations is more likely to elicit an appropriate public response and may increase public awareness. In addition, confusions can be reduced, mutual understanding between public and responders can be improved, and decision capabilities can also be improved. These recommended improvements in communication need to be tested before implementation.

#### Insurance management

By having an overall picture of each disaster and its potential severity level, the UDSCS will help insurance agencies and estimators to create specific criteria to clarify common disaster compensation packages and insurance policies (Caldera et al. 2016b).

#### Information management

Information managers can use the clear terms outlined in the UDSCS to improve the poor quality of the data in the existing reporting databases. Easily recognizing an event occurrence and having a set of standard terms in a proposed UDSCS is expected to allow database managers to improve information management and processing. A standardized database terminology and the associated data can be managed to mitigate missing or inaccurate data. Using common terminology to clearly identify the scale of a disaster can be the standard used to record disasters. Then, the scale can be used to record global disasters and the subdivisions of continental, regional, and national records. Common terminology can also be used to record joint disaster records (i.e., combined impact of primary and secondary disasters), and separate disasters can be recorded as subdivisions of the records, where possible (if the impact of primary and secondary disasters can be separated clearly). As a result, complications, misunderstandings, misclassifications, and missing records can be minimized as much as possible. Additionally, decision capabilities of disaster information management processing can be improved as this universal system classifies disasters according to severity.

#### Research community

The UDSCS has an academic value in addition to practical applications. For example, if we have an accurate disaster database, more research can be conducted on disaster mitigation to improve disaster preparedness technologies. It may take many years to obtain quality reports. However, even relatively short records can be used to develop relationships among variables in the records in databases (Brooks 2013), which improves analysis and research.

### Improved communication

The UDSCS will serve as a bridge between qualitative and quantitative techniques used in emergency management systems. Qualitative and quantitative techniques are integrated in the UDSCS to produce management and size measurement systems, respectively. Therefore, the UDSCS is expected to avoid inconsistencies and, most importantly, connect severity metrics to generate a clear understanding of the degree of an emergency and the potential impacts, thereby improving mutual understanding between the emergency management systems of countries at all levels: international, continental, regional, national, provincial, and local.

## Discussion

As UDSCS is used post-event, the classification of the severity of the event may change as reports on the number of fatalities are updated. Therefore, the degree of severity changes with time and with updated reporting on the disaster. For example, an earthquake, which occurs in seconds, could be categorized as a “disaster” in terms of severity within the first few hours depending on the reported impacts and causalities. However, the impact and causalities can increase days or weeks after the event. Accordingly, the severity of the earthquake could be reclassified as a “calamity” a day or two after the event, and it could potentially be considered a catastrophic event within weeks. Although frequent updates improve the accuracy of the severity, it is vital to estimate the severity shortly after an event strikes to provide information to first responders and for public reporting and planning. The potential impact of a disaster can be estimated with a certain degree of accuracy, which is beneficial because the size of a first-responder contingency depends on the magnitude of the disaster impact. Therefore, predicting the severity can accelerate the recovery process.

The information in the initial UDSCS, listed in Table 13, is proposed for the first time. The most important advantage of the UDSCS is that it provides a consistent method for all stakeholders to measure the severity of all types of disasters. A common scale is more informative than the variety of scales currently used for different disaster types and for different stakeholder groups because the classification applies to all types of disasters and all stakeholder groups.

In addition, the UDSCS has a reasonable and standard number of levels to articulate the full range of disaster severity, and it has a clear order of seriousness for the severity levels. The increasing level of seriousness from 0 to 10 is defined using quantitative boundaries and clearly defined descriptive terms, which avoids confusion as to whether UDSCS 0 or UDSCS 10 is the most critical. Because the UDSCS has a reasonable number of levels, events that have different levels of severity will not be in the same category. Therefore, because this universal measurement system clearly conveys the size of the impact of a disaster, it avoids confusion and improves mutual understanding among stakeholder groups.

Moreover, the UDSCS can be adapted to any language, country, or culture. The UDSCS clearly defines the levels of the disaster continuum by (1) redefining the existing terms without using one term to define another, (2) outlining the impact factors, damage, injuries, and fatalities, and (3) using better descriptive words to reflect the order of seriousness of a disaster.

The number of fatalities is chosen as the most influential factor because it is correlated with several factors that affect humans. Fatalities is highly correlated with injuries and missing persons and moderately correlated with houses damaged and cost of damage. However, one factor alone is not sufficient to measure the severity of disasters because a single factor does not address all aspects of severe events. For example, a disaster, such as a wildfire in an uninhabited forest, may affect only a geographical area and not have any direct and immediate impact on humans, but the wildfire may have long-term adverse effects on the local and global ecosystems. Real-world examples include the 2016 Fort McMurray fire, which had no fatalities, and the 2013 Alberta flood, which had 4 fatalities, but both disasters were the costliest Canadian disasters in history; consequently, neither event is properly represented using a scale that only considers fatalities. Therefore, a more advanced multidimensional quantitative scale that combines all impact factors, such as fatalities, injuries, homeless, affected population, area affected, and cost of damage, is needed to properly address the full range of a disaster impact.

Even using one impact factor, this simple universal system that incorporates all types of natural disasters (rather than the variety of unrelated scales for specific disasters) is more informative and consistent for assessing severity. The boundaries of the levels are clearly defined. Therefore, an overall picture of the disaster continuum is available using the UDSCS. In addition, the UDSCS links the disaster severity matrices because it serves as a bridge between quantitative and qualitative techniques.

This research was completed mostly prior to coronavirus pandemic in 2019–2020 (COVID-19); therefore, COVID-19 is not discussed in detail in this paper except in this paragraph. COVID-19 is an acute respiratory infectious disease that affects humans and some animals, and it is caused by the 2019 novel coronavirus (2019-nCoV); the first patient to be infected is unknown (Zheng et al. 2020). However, it first appeared in Wuhan, China in December 2019. During the initial phase of this virus, Chinese doctors and scientists issued warnings of a global pandemic (Huang et al. 2020). The World Health Organization (WHO) announced that the outbreak of COVID-19 is a global pandemic on 12 March 2020 (World 2020). Different countries have used different methods to control the spread of COVID-19. Tragically, this outbreak rapidly escalated from endemic to epidemic within a few days and from epidemic to pandemic within a few months (Centers 2020), and numerous new COVID-19 cases are reported daily around the world. As of 08 February 2021, there have been more than 106.7 million confirmed cases and more than 2.3 million fatalities reported globally (Johns 2021). According to the current numbers COVID-19 is categorized as a Catastrophe Type 2 (UDSCS 7) event.

## Conclusions

The novel Universal Disaster Severity Classification Scheme (UDSCS) is developed to assess the impact of any uncontrollable forces of nature regardless of disaster type, place, or time. This universal severity classification system is applicable to all stakeholders, such as civilians, emergency responders, disaster managers, relief agencies, all levels of government, NGOs, insurance managers/estimators, reporters, media, database/information managers, academics, researchers, and policy makers. Therefore, it is expected to create a universal standard severity measurement system, and most importantly, generate a common communication platform to describe the impact of disasters, ensuring mutual understanding across the globe. A nation’s ability to prepare and manage extreme global disasters that affect more than one country will improve if there is mutual understanding among different countries’ emergency management systems at all levels.

By selecting the appropriate terms for the levels and naming the categories using plain language to describe the magnitude of a disaster, the UDSCS is expected to allow for easier management at all levels. Moreover, combining these terms with quantitative techniques gives clear boundaries and guidelines, and combining these terms with the color coding scheme enables easy adaption to any language, country, or culture. The color coding system is helpful to some people working or involved in disaster recovery who are not literate or cannot understand the local language or dialect (if working in foreign regions). Therefore, the definitions and colors together ensure broader communication between people and organizations.

The UDSCS explains the disaster continuum. Using this universal system, the impact of a broad range of natural disasters that occur anywhere in the world at any time can be described, measured, compared, assessed, and ranked both quantitatively and qualitatively. The UDSCS uses a color coding scheme and disaster terminology to describe disasters qualitatively, and it uses severity levels and impact factor boundaries to assess disasters quantitatively using the rating scale 0–10 to rank disasters. Further, it uses the probability of occurrence of extreme disasters to predict the impact of any natural disaster. Most importantly, the UDSCS is a single common measurement for all types of natural disasters because it integrates colors, words, impact factors, and severity level rank.

The proposed severity scheme will improve communication and understanding of disaster risks, which aligns with the priority of the Sendai Framework for Disaster Risk Reduction 2015–2030. Additionally, the UDSCS is a simple scientific instrument. The selected descriptive terms, impact factors to measure severity, and proposed ranges are based on data and statistically robust. Furthermore, the UDSCS will avoid inconsistencies and, more importantly, will connect severity metrics to generate a clear understanding of the degree of an emergency and the potential impacts. Lastly, qualitative and quantitative techniques are integrated to produce management and size measurement systems, respectively.

## Future extensions

This is an ongoing research project to develop a multidimensional UDSCS to understand the disaster continuum. The scope of this paper is to introduce an initial UDSCS that can be used to compare the impact of any type of natural disaster both qualitatively and quantitatively. When developing quantitative measures (in Step 4), we considered only one impact factor, fatalities, to develop the initial UDSCS. However, using the initial scale with one factor does not capture all aspects of an impact, as noted previously. Therefore, an advanced multidimensional scale that combines all impact factors using a disutility function needs to be developed.

## Declarations

### Availability of data and material

The data that support the findings of this research are as follows: All types of natural disaster data from EM-DAT, the international disasters database of the Centre for Research on the Epidemiology of Disasters (CRED), Brussels, Belgium. www.emdat.be. Tornado data from the Storm Events Database, National Oceanic and Atmospheric Administration (NOAA) National Centers for Environmental information. http://www.ncdc.noaa.gov/stormevents/. Volcanic eruptions data from the National Geophysical Data Center /World Data Service (NGDC/WDS): NCEI/WDS Global Significant Volcanic Eruptions Database. NOAA National Centers for Environmental Information. https://doi.org/10.7289/V5JW8BSH.
